# Tackling the technological, environmental, legal, and economic challenges of ocean alkalinity enhancement: A case study from the Baltic sea

**DOI:** 10.1016/j.isci.2026.116928

**Published:** 2026-07-30

**Authors:** Anu Lähteenmäki-Uutela, Jáchym Judl, Jari Silander, Sonja Lavikko, Tuukka Mäkelä, Carolin Regina Löscher, Ulf Riebesell, Markus Latvala, Kristian Spilling

**Affiliations:** 1 Finnish Environment Institute, Helsinki, Finland; 2 Geological Survey of Finland, Espoo, Finland; 3 National Bureau of Investigation, Vantaa, Finland; 4 University of Southern Denmark, Odense, Denmark; 5 GEOMAR Helmholtz Centre for Ocean Research, Kiel, Germany; 6 Feasib Ltd. Finland, Ylivieska, Finland; 7 Centre for Coastal Research, University of Agder, Kristiansand, Norway

**Keywords:** ocean alkalinity enhancement, mining side product, environmental impacts, legal analysis, life cycle assessment, cost-benefit analysis

## Abstract

Ocean alkalinity enhancement (OAE) is a possible climate mitigation technology that can remove CO_2_ from the atmosphere. Here, we studied the feasibility of OAE in a case study using serpentinite gangue and examined the carbon dioxide removal (CDR) potential, the legal requirements, and the economic viability. The carbon emission factor was estimated to stay below 70 kg CO_2_e t^−1^ CO_2_ removed. Permitting would require the risks for marine ecosystems at the site to be carefully considered. Under the assumptions of this case study, OAE would require CDR credit prices higher than 100 € t^−1^ CO_2_ removed to be economically viable. Commitments of public and private actors to buy OAE credits would reduce market uncertainty and could be a way to promote OAE if the environmental impacts are acceptable.

## Highlights

• A multidisciplinary ocean alkalinity enhancement case study
• Environmental permit depends on the ecological impacts of contaminants
• The carbon emission factor of the case study was 40–70 kg t^−1^ CO_2_ removed
• The economic costs were estimated to 90€–200€ t^−1^ CO_2_ removed

## Graphical abstract

**Figure:**
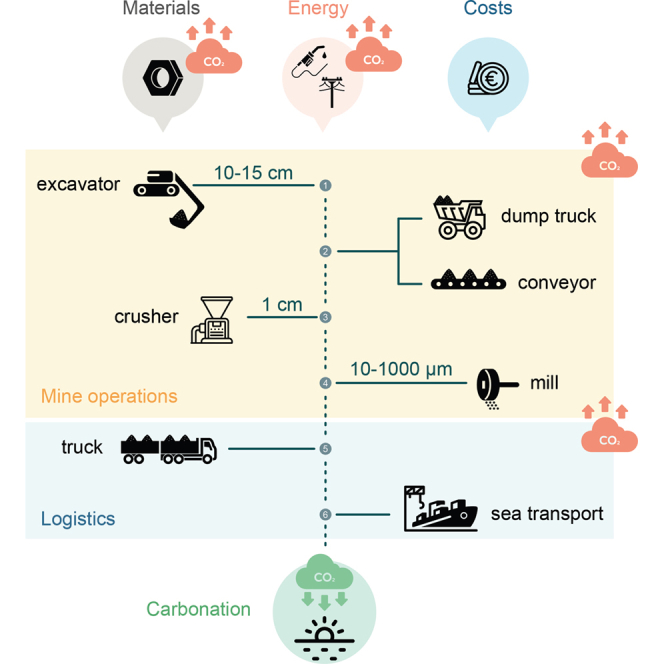

## Teaser

Earth sciences; Environmental geochemistry; Marine geochemistry; Environmental science; Aquatic science; Oceanography

## Introduction

CO_2_ concentrations are rising at an unprecedented pace, threatening the habitability of our planet. Thus, urgent action is required to slow down CO_2_ emissions and remove CO_2_ from the atmosphere.^1^ There are different carbon dioxide removal (CDR) approaches, one of which is to enhance the weathering of rock.^2^ Rock weathering has been a key factor in temperature stabilization on Earth on geological time scales,^3^^,^^4^^,^^5^ and enhanced weathering could facilitate temperature stabilization. Rain, wind and waves slowly erode rock that contains minerals, and some of these minerals react with CO_2_ to form carbonate compounds. This reaction naturally removes ∼1.5 Gt CO_2_ per year, but it is too slow to counteract the current human release of ∼38 Gt CO_2_ per year.^6^ However, it is technically possible to speed up the weathering process by grinding up minerals, providing more surface area to react with CO_2_.^7^^,^^8^ On land, such human intervention is termed enhanced weathering. When the minerals are distributed into the ocean and reacting with CO_2_ dissolved in seawater, the process is called ocean alkalinity enhancement (OAE). Increasing alkalinity will enhance the ocean’s uptake of CO_2_, forming mainly bicarbonate.^9^ The two main groups of minerals that could be used for OAE are silicate minerals (e.g., olivine) or carbonates (e.g., limestone), and the alkalinity-enhanced ocean thus operates as a larger-than-before carbon sink.^10^

The carbonate chemistry and theoretical potential of OAE have raised a lot of scientific interest during the past decade.^11^^,^^12^^,^^13^^,^^14^ In theory, global mineral reserves could be used to remove thousands of Gt of CO_2_.^15^ According to Caserini et al.,^16^ 6.6 Gt of materials would be available per year. For the net removal of 1 Gt of CO_2_ through OAE, 1–3 Gt of OAE material is theoretically needed. For comparison of scale, iron and copper mining combined extracts ∼12 Gt of source material yearly.^17^ A less costly alternative to mining is to use already mined and ground materials. Suitable mine tailings are distributed globally with the potential to remove up to ∼0.5 Gt CO_2_ per year.^18^

There are still knowledge gaps, barriers or challenges that have hindered the implementation of OAE projects. The net carbon impact is central; OAE must provide a verified net carbon offset for the whole process that could be sold, including the mining, crushing, grinding/milling, transportation and distribution of the minerals. All steps require energy and need to be accounted for in the case-specific carbon balance (ISO 14067).

The energy consumption of milling is critical. Calculations with olivine show that milling down to 1 mm causes climate impacts of 14.2 kgCO_2_e t^−1^ CO_2_ removed, while milling down to 10 μm, or even to 1 μm, particles increase the carbon emission factor to 51 kgCO_2_e or over 200 kgCO_2_e, respectively,^19^ based on Bond’s crushing index. The optimization of fine milling can save energy, up to 30%–40%.^20^

Milling is also central for the CO_2_ removal impact and its timing. Reducing the particle size of the mineral increases the dissolution rate and hence the reaction with CO_2_. The full capacity of CO_2_ removal may take up to 100 years to develop, but a more optimistic scenario is that OAE can reach carbon negativity within a year.^21^ At the same time, only particles below 10 μm can reach the full theoretical impact over time and local conditions such as temperature affects dissolution rates.^21^ In addition, the risk of triggering secondary precipitation or other alkalinity loss, e.g., by biological alteration, must be considered in the carbon balance.^22^^,^^23^

Reducing transportation distance saves energy, but depends on the geographic distribution of materials, for example, less than 5% of suitable calcium carbonate deposits are within 10 km of the coast, and 25% within 100 km and nearly 50% of carbonate rocks suitable for OAE are in Africa.^16^ The use of fossil-free energy and electric vehicles improves the carbon balance.^24^^,^^25^

The risks of large-scale OAE operations for the marine environment have major knowledge gaps with both general and case-specific concerns.^26^ The environmental risks of OAE are evaluated in environmental permit procedures.^27^ Under the United Nations convention on the law of the sea (UNCLOS), coastal states have jurisdiction in their territorial waters, extending 12 nautical miles from their coastlines, and over their exclusive economic zone (EEZ), extending 200 nautical miles from their coastlines. As shallow coastal waters are the likely environment for OAE activities,^28^ coastal states will typically have jurisdiction, and national environmental and water laws of each coastal state apply. International law is relevant as national laws of the coastal state must align with the international law commitments of that state. The London convention (the convention on the prevention of marine pollution by dumping of wastes and other matter, in force since 1975, LC) as well as the later adopted London protocol (in force since 2006, LP) relate to placing matter in the sea.

In the permitting of OAE, the scale, the location, and the materials used are decisive for whether a permit is required and granted, and which precautions are required under the permit. Operators and authorities must understand the chemistry of different OAE minerals and their potential impacts on marine ecosystems. Candidate minerals for OAE include quicklime CaO and hydrated lime Ca(OH)_2_,^29^ olivine (Mg,Fe)_2_SiO_4_, a magnesium iron silicate,^22^ and serpentinite Mg_3_Si_2_O_5_(OH)_4_, a magnesium silicate.^30^ Olivine and serpentinite represent different stages of the ultramafic rock life cycle, and feature very different mineralogical characteristics. Olivine is a primary, anhydrous mineral with high Mg content; serpentinite is a secondary, hydrous rock produced by hydration of olivine and pyroxene. These differences crucially affect their reactivity and have important implications for OAE. Pure forsteritic olivine offers a higher theoretical alkalinity yield per unit mass and can sequester more CO_2_, but dissolution rates in seawater are slow. Serpentinite contains less reactive serpentine minerals (i.e., lower potential for CO_2_ removal) but may contain brucite, which can release alkalinity at a higher rate than olivine.^31^

There seems to be relatively little effect of OAE on primary producers,^32^^,^^33^ but silicate-based OAE could shift the community composition of marine primary producers as some groups of microalgae (e.g., diatoms) use silicate whereas most other groups do not.^13^ Optimization of injection and distribution methods helps in reducing the negative impacts including the precipitation of calcium carbonate CaCO_3_.^33^ There could be potential disruption in biogeochemical cycles with large scale OAE,^34^ as changes in surface water biota may indirectly impact deep-sea biota, and the organic matter flux.^35^^,^^36^ Other important and possibly more relevant factors for marine life include trace metal toxicity, sudden pH changes which might kill organisms, shadowing, or causing blooms by iron additions, which might sink out and lead to high O_2_ consumption in shallow coastal waters. The biggest threat comes from secondary elements that would also be dissolved in connection with doing OAE, such as nickel, chromium, and cadmium, but this will depend on the source material used.

In addition to the technology and the ecology, the third major sphere of uncertainty lies in the economic basis for OAE; a payer is needed, and income must exceed costs.^37^ Buyers of CDR credits could be public and/or private, including states,^38^ cities, companies and individuals that either by duty or voluntarily target a specific carbon balance. In theory, removal companies and emitting companies should operate in the same markets.^39^^,^^40^^,^^41^The emission cap set by regulators affects the prices in compliance markets; prices rise if operators struggle to match the cap. States can also intervene markets by buying offsets or by providing direct investments, public loans, R&D grants or tax incentives.^42^^,^^43^ Honegger et al.^44^ and Buck^45^ see that CDR might best be framed as a public good or service, i.e., a service that states pay. CDR credits (for verified CO_2_ removal) must typically be certified and fulfill criteria such as permanence and additionality (e.g., EU carbon removals and carbon farming regulation EU/2024/3012). For the OAE service seller, not only the costs of the physical operations but also the certification, registration, and validating costs must be covered by the offset price.^44^ Globally, a handful of blue carbon projects have sold carbon offsets, based on mangrove forests and seagrasses sequestering carbon.^41^^,^^46^ Social, governance, financial, technical, and scientific barriers and uncertainties have slowed down wider operationalization.^41^^,^^47^ Reliable settings,^48^ the protection of marine ecosystems^41^^,^^49^ and a reasonable price would make OAE attractive to investors and buyers.^47^

Here, we look at the technological, environmental, legal/governance, and economic perspectives of an OAE operator using a concrete case study of serpentinite gangue dispersal in the Baltic Sea, with the questions.

• How can we ensure the CO_2_ removal through the OAE process?
• How to tackle the risks for the marine ecosystems and the associated legal barriers?
• How could OAE be economically profitable (income exceeding costs)?

To answer these research questions, we performed a life cycle assessment (LCA), a desk-study environmental risk assessment, a legal analysis, and a cost benefit analysis, each based on the characteristics of the case. We demonstrate how the challenges in the hypothetical case could be tackled. From that we draw general-level conclusions on the barriers and knowledge gaps for OAE and point to the uncertainties in the ecological and carbon impacts, which translate directly into legal and economic uncertainties in society.

## Results and discussion

### Description of case study

**Figure 1:**
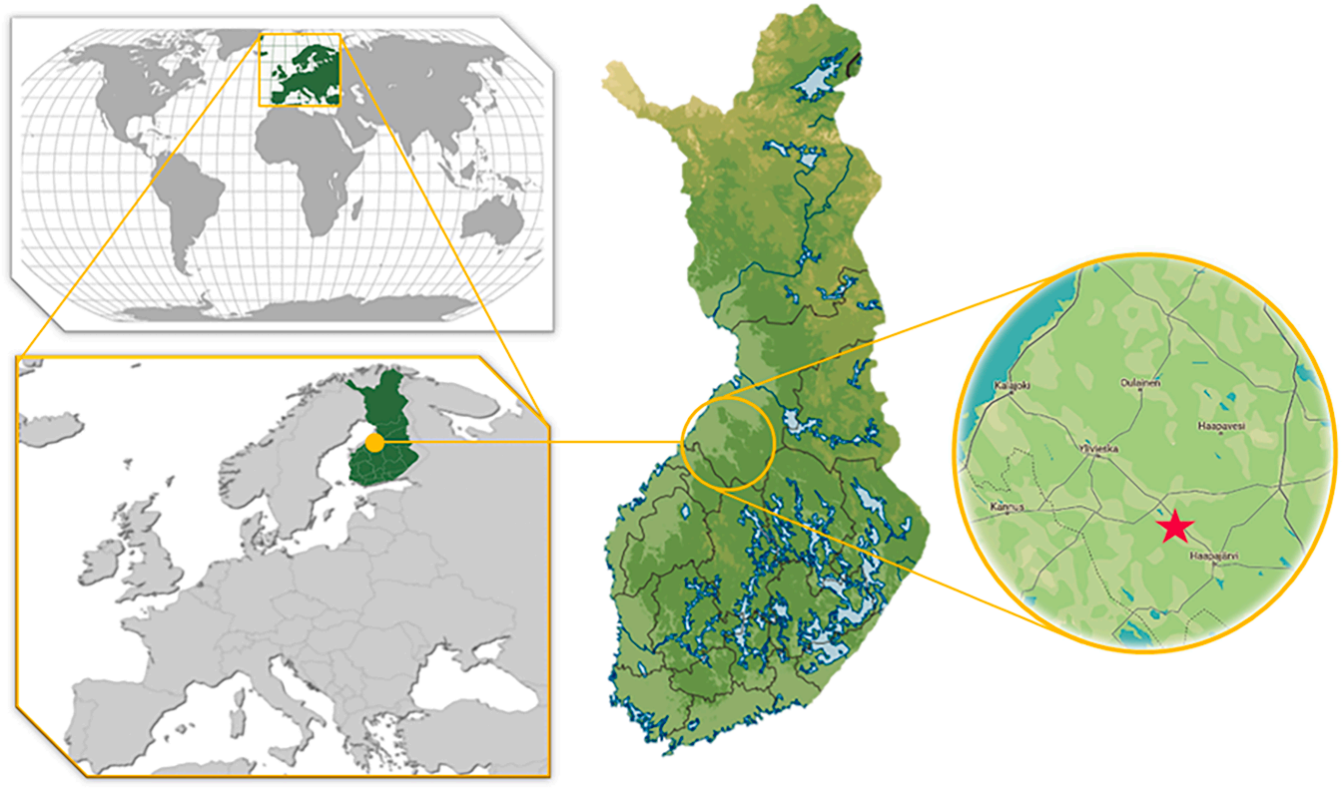
Location of the Hitura mine

Here we looked at the materials from the closed Hitura nickel mine in Finland, located 400 km north of Helsinki (Figure 1). Hitura's landscaped serpentinite area covers 11.5 ha and contains 2.2 million m^3^ of rock, or approximately 5–6 Mt of gangue suitable for OAE. Compared to mining pristine rock, using gangue for OAE may have higher net carbon reduction potential, cost less, and avoid additional negative landscape and ecosystem impacts.

The Hitura mine was in active operation from 1965 to 2013 and decommissioned in 2015. Finland’s Environmental Protection Act and the environmental permit for the operations required the waste areas to be covered and the landscape to be restored. The close-up and landscaping operations from 2019 to 2021 covered a total area of 140 ha for gangue and tailings. The cost of closing the mine was 18.5 M€ at the end of 2021, and environmental monitoring and wastewater treatment continue to increase the costs: for the years 2024–2027, 1.6 M€ per year has been reserved for preventing environmental damages.^50^ As the original mining company went bankrupt before the clean-up in 2015, the costs are covered by the state of Finland. Here, we do not propose to open the already landscaped mine; we rather ask whether using similar cases for OAE could be a sensible CDR option in the future.

In our hypothetical case scenario, the gangue is ground and milled at the Hitura site, transported to the nearest harbor (100 km by road) and 12 nautical miles or 22 km off the coast to where the Finnish EEZ begins,^18^ and dispersed in the Bothnian Bay, the northernmost part of the Baltic sea, where the average depth is 43 m.

The Hitura ore body mainly consists of serpentinite (Mg_3_Si_2_O_5_(OH)(_4_) that reacts with CO_2_ and water, leading to an increase in the alkalinity of seawater. Serpentinite is in general a good material for mineral carbonation, and the Mg-rich lizarditic variety of serpentine, descending from olivine, as the most promising.^51^ The potential of Hitura serpentinites for OAE has been evaluated.^51^^,^^52^ The serpentinite in the Hitura ore body is not a homogenous entity; various serpentine minerals and serpentinites have been identified.^53^ In addition to serpentinite, Hitura has lhertzolite, mica gneiss, and amphibolites,^54^^,^^55^ which are not suitable for hydrous carbonation due to their crystal structure or other mineralogical characteristics.^56^ Hitura serpentinites as its other rocks carry metals and sulfides,^55^^,^^57^ the release of these needs to be carefully assessed. Hitura has both gangue and tailings. Tailings are in readily ground form, however, the chemical residues from the enrichment process (e.g., Ca(OH)_2_, sulfuric acid, xanthates, acetates, CuSO_4_) may cause environmental hazards.^53^ The concentrations of heavy metals in Hitura tailings exceed the upper guideline values for contaminated soil (Co, Cr, Cu, and Ni, in sulfide phase mostly). Therefore, we excluded tailings and here only focus on the serpentinite gangue.

### LCA: Reaching a net carbon removal with serpentinite gangue in the Baltic sea

The dissolution time of serpentinite gangue has not been extensively studied under a natural environment, but likely take several years, but smaller grain size, more physical forcing (wind driven mixing), increased temperature, and reduction in pH will increase the dissolution rate.^21^ Wind driven mixing reduces the dissolution time by reducing the diffusive boundary layer around the particles.^58^ In the shallow case study area (under 50 m deep), larger particles would sink to the seafloor where sediments are mostly clay (a homogenous layer). Sinking down to the sediment likely slows down dissolution, but there could still be alkalinity released at the seafloor, and with a relatively shallow depth the water column is fully mixed annually.

Based on Ramasamy et al.^21^ on olivine, and taking the environmental conditions into consideration, the particle size would have to be smaller than 1 mm for serpentinite to have a meaningful OAE contribution in the Baltic sea, although larger particles will only partly dissolve. We defined three distinct scenarios for particle sizes 10 μm, 100 μm, and 1000 μm to gain an understanding of the relationship between particle size and carbon impact of the system. Other variables were kept constant across the scenarios.

**Figure 2:**
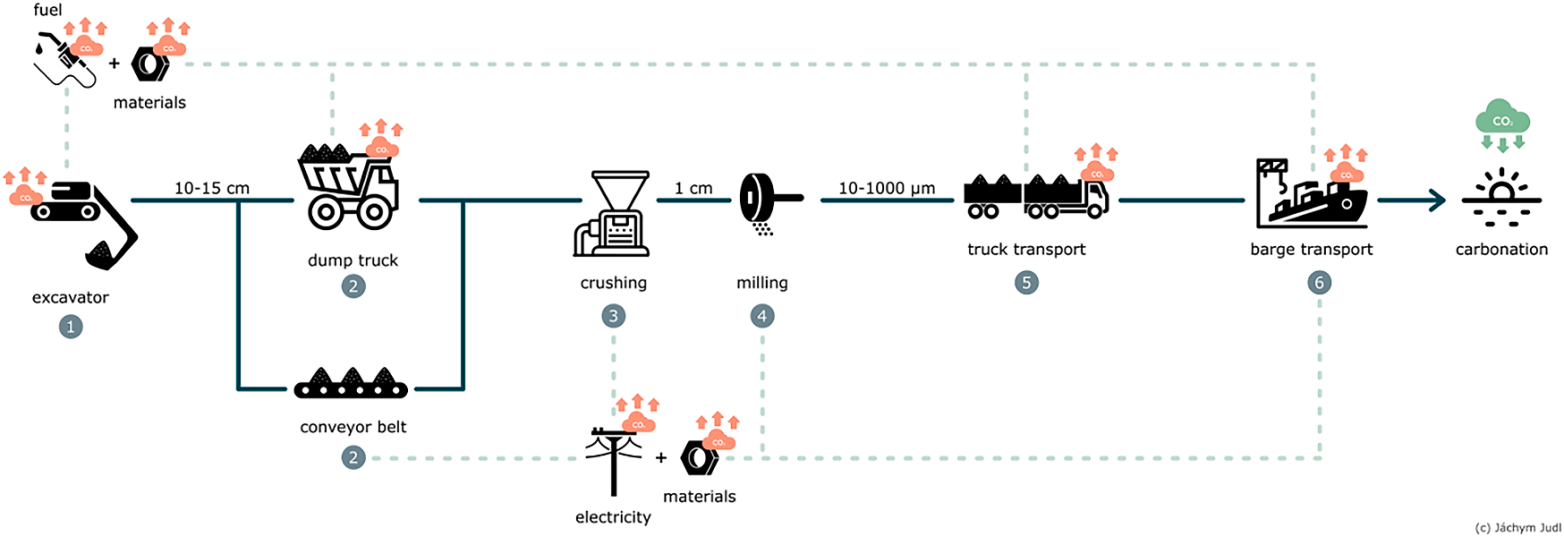
An outline of the studied product system and processes included in the analysis

The analyzed system includes all relevant activities along the value chain of finely milled serpentinite rock from Hitura as it would have been utilized before the mine closure and landscaping (Figure 2). It includes the following activities: (1) excavator operations; (2) transport of the serpentinite gangue (10–15 cm rocks in diameter) on-site to the crusher either on a dump truck (∼600 m) or on a conveyor belt; (3) crushing of the rock, reducing the size to ∼1 cm; (4) milling the rock to powder of various dimensions (10–1000 μm); and (5) truck transport of the powder to the port of Kokkola (100 km); and pontoon or barge transport (22 km) to the sea where it is discharged and where carbonation occurs over time.

Mineral dispersal in the sea should be guided by understanding dissolution kinetics and the efficiency of CO_2_ uptake. Mineral dispersal rate is often modeled in the range of 10–20 t km^−2^ d^−1^.^59^ At this dispersal rate, a small barge with a capacity of 3000 t, and operating speed of 5–8 knots would require approximately 2–5 days to discharge a full load. By avoiding the ice-covered winter months, operations could continue for 6–8 months per year, allowing two barges to distribute ∼0.5 Mt of material annually. Larger dry bulk carriers could carry larger loads and could cover larger areas at increased speeds. The available material from the Hitura mine (∼5 Mt) would be sufficient for 10 years of operation at 0.5 Mt yr^−1^ with a barge operation, or all in a single year with dry bulk carriers. Assuming an optimistic 0.4 carbonation efficiency (t CO_2_ removed per t gangue dissolved), this would correspond to the removal of 0.2 Mt CO_2_ per year of operation with barge or 2 Mt CO2 with dry bulk carriers. This would compensate for ∼0.3% or 3% of Finland’s annual fossil CO_2_ emissions, respectively. The relatively low application rate of 10–20 g m^−2^ would likely help avoid excessive concentrations of contaminants such as nickel, which are present in serpentinite.^60^

**Figure 3:**
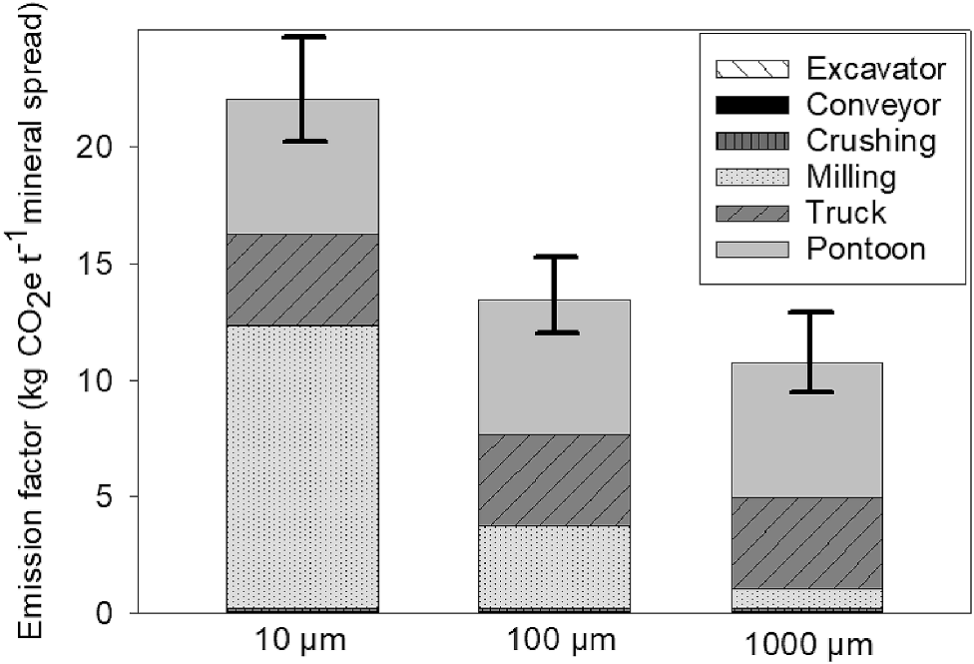
Life-cycle assessment results of ocean alkalinity enhancement (OAE) using three different milling sizes Climate impacts, was calculated using the EF 3.0 method with IPCC AR6 (2021) characterization factors (GWP100), are reported per metric tonne of mineral spread in the ocean. Error bars indicate the uncertainty range (95% confidence interval) based on Monte Carlo simulation (5000 iterations).

Our scenario results show that the particle size has a significant influence on the emission factor of the milling/grinding process and the whole system (Figure 3). The finer the particles are ground, the more electricity the mill consumes, which results in higher greenhouse gas emissions. The calculated carbon emission factor for the three scenarios (10 μm, 100 μm, and 1000 μm) with fixed parameters are 22.06 kgCO_2_e t^−1^ of ground mineral, 13.48 kgCO_2_e t^−1^, and 10.77 kg CO_2_e t^−1^, respectively. Depending on the scenario, either milling or the sea transport of the milled product are primary contributors to the overall carbon footprint with contributions of 8%–55% and 26%–54%, respectively. Truck transport of the milled product from the mine to the harbor contributes between 18% and 36% depending on the scenario. The other processes contribute insignificantly to the overall results, with contributions of around 1%, or almost no contribution at all (excavator or mine transport).

The Monte Carlo simulation results indicate a range of carbon emission factors caused by the value chain (Figure 3). Increasing uncertainty correlated with decreasing particle size. Within the 95% confidence interval, the impacts of the 10 μm scenario range between 20.3 and 24.7 kg t^−1^ of rock (median value of 22.1 kg), for the 100 μm scenario 12.3 and 15.4 kg (median 13.5 kg), and for the 1000 μm scenario 9.7 and 12.7 kg (median 10.8 kg). The main parameter influencing the uncertainty is the Bond Work Index of the rock (10–30 kW h t^−1^, default 20 kW h t^−1^).

**Figure 4:**
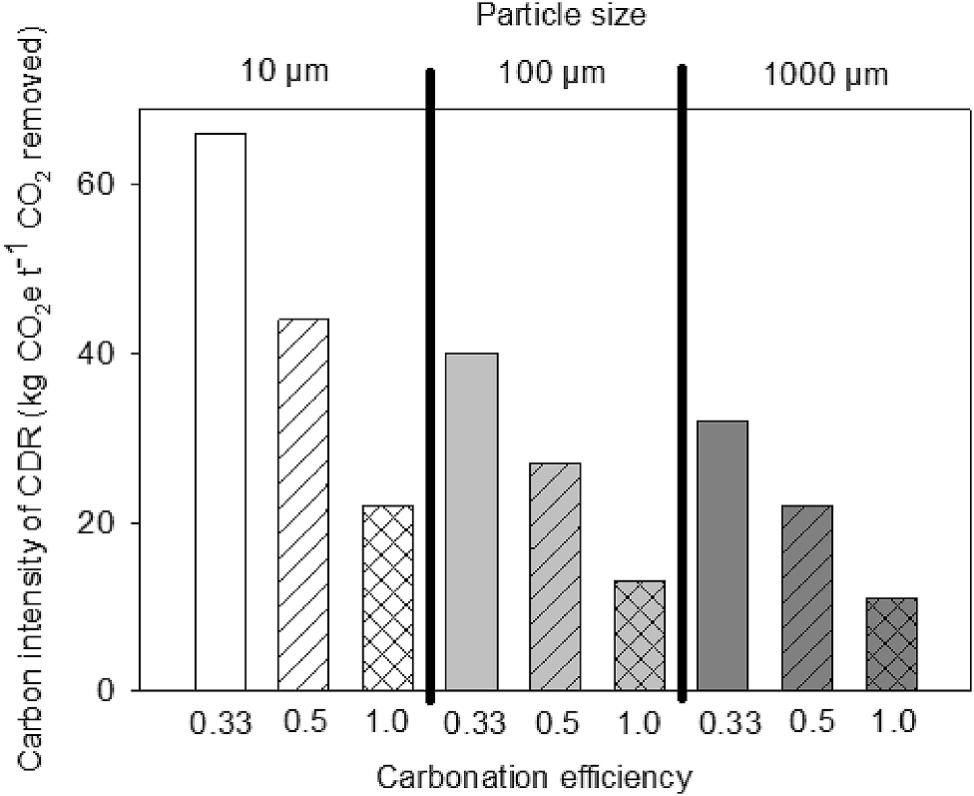
Influence of particle size and carbonation efficiency on the carbon intensity of CO_2_ removal (CDR) of ocean alkalinity enhancement (OAE) Climate impacts are reported per functional unit of net carbon dioxide removal (tCO_2_ removed), calculated using the EF 3.0 method with IPCC AR6 (2021) characterization factors (GWP100). Results illustrate the combined effect of milling size and carbonation efficiency on the net climate performance of the system.

For understanding life cycle CO_2_ emissions per t CO_2_ removed, carbonation efficiency is decisive (Figure 4). Carbonation efficiency is complex, depending on factors, such as temperature, pH, and reaction surface area, i.e., particle size.^21^ In the Baltic sea, the carbonation efficiency for olivine is 0.17–0.46 tCO_2_ removed t^−1^ olivine used depending on the season.^21^ Higher efficiencies, close to 1.0, have been obtained but should be viewed as a best-case scenario, perhaps achievable only with grain sizes below 1 μm.^61^ For the LCA, we compared three scenarios based on carbonation efficiencies 1, 0.5, and 0.33 meaning that we consider the reference flows of 1, 2, or 3 t of mineral to provide the same functional unit of sequestering 1 t of CO_2_.

The value chain GHG emission depends on the particle sizes and carbonation efficiencies (Figure 4). In practice, larger particle sizes (e.g., >10 μm) and lower effective CO_2_ uptake efficiencies (e.g., 0.3–0.5) may be more realistic for serpentinite gangue deployed in relatively cold environments such as the Baltic sea.^11^ In all scenarios OAE was carbon negative (Figure 4), and the potential climate footprint of sequestering 1 tCO_2_ with carbonation efficiency 1 was estimated to be in the range of 11–22 kg CO_2_e t^−1^, or 9.73–24.7 kg CO_2_e t^−1^ if model uncertainty is considered (Figure 3). Taking carbonation efficiency into account (Figure 4), it is likely that the carbon emission factor per functional unit of 1 t of sequestered CO_2_ would stay below 50 kg CO_2_e with a carbonation efficiency 0.5, and below 70 kg CO_2_e with carbonation efficiency 0.33, in line, e.g., with Foteinis et al.^19^

### Tackling the environmental risks and legal issues

The main result of the legal analysis was that the operator of large-scale OAE can and must apply for an environmental permit. As per UNCLOS, the Finnish national jurisdiction extends to territorial waters, extending 12 nautical miles off the coastline, and to the EEZ, extending 200 nautical miles off the coastline. Finland regulates OAE activities in this area. Permit decisions will be based on Finnish national legislation and the international conventions signed and ratified by Finland. Finland is bound by the LC and the LP as well as the Helsinki convention (convention on the protection of the marine environment of the Baltic sea area), all of which prohibit dumping.^62^ A 2013 amendment to the London protocol (Resolution LP 4(8)) established a framework for regulating marine geoengineering activities, initially focusing on ocean fertilization.^63^ The amendment has been accepted by Finland and five other states, but it is not in force. The slow acceptance of the 2013 amendment has been described as a matter of concern in recent IMO meetings.^64^ In this section, we focus on the existing legal framework.

LC signatories agree to “promote the effective control of all sources of pollution of the marine environment” (Article 1). LP goal is “to protect and preserve the marine environment from all sources of pollution” and “to prevent, reduce and where practicable eliminate pollution caused by dumping or incineration at sea of wastes or other matter” (Article 2). Dumping is “the deliberate discharge into the sea of wastes and other matter” (Convention Article 3, Protocol Article 1). The contracting parties are required to regulate dumping in their offshore areas, from vessels or aircraft registered in their territory, and from vessels or aircrafts loaded within their territory (LC Article 7; LP Article 10). LC Annex I has a blacklist of wastes and other matter, the dumping of which is prohibited (Article IV(1) (a)). The blacklist includes for example mercury, cadmium, oil, high-level radioactive materials, and persistent plastics. Another category of substances (Annex II) requires a special permit issued only after careful consideration (Article IV(1) (b), Article VI(1) (a).) These include wastes containing significant amounts of arsenic, lead, copper, or zinc, including their compounds. When dumping large quantities of acids or alkalis, consideration shall be given to possible presence of beryllium, chromium, nickel and vanadium, including their compounds (LC Annex II). The LC does not prohibit the dissemination of alkaline substances as such. The LP, in turn, prohibits the dumping of all wastes and other matter that is not on its reverse list. The reverse list of substances that may possibly be allowed includes “inert, inorganic geological material” (LP Annex). This means LP parties may consider permitting OAE, if general precautions are followed.

Alternatively to reading the LC and LP Annexes, OAE can be considered as not dumping in the first place, as dumping excludes “placement of matter for a purpose other than mere disposal thereof, provided that such placement is not contrary to the aims” of LC and LP.^27^^,^^65^

By signing the LC and LP, Finland has agreed to regulate all action within its jurisdiction that may pollute marine environments. The Finnish marine protection act (1415/(1994) prohibits the pollution of international waters and applies to Finnish citizens and ships sailing under the Finnish flag. Dumping is defined as “intentionally submerging or otherwise discarding waste or other material into the sea from any type of vessel (Section (4) and prohibited (Section 7). Section 18.1. of the Finnish environmental protection act (527/2014), in turn, prohibits pollution in the Finnish territorial waters and in the Finnish EEZ. Dumping of “waste and other material” … “to be submerged or otherwise discarded” is prohibited (Section 18.2.).

We asked the Finnish authorities if OAE is “dumping other materials to be submerged or otherwise discarded”. We asked the ELY (Elinkeino-, liikenne-ja ympäristökeskus, Center for economic development, transport and the environment, the authority that assessed the need for permit until end of 2025), AVI (Aluehallintovirasto, regional state administrative agency, the permitting authority until end of 2025), and the ministry of environment. The ministry referred to the LC and LP obligations of Finland, highlighting section 1 of the LP: dumping is not: “placement of matter for a purpose other than the mere disposal thereof, provided that such placement is not contrary to the aims of the present protocol”. As the purpose of OAE is not disposal, OAE is considered possible through permitting. The permitting authority (AVI) would need information on the substance and its impacts in the marine environment, based on which it would make its decision. If the operations were contrary to the aims of the LP, the operations would be considered as dumping, and a permit would not be granted.

In one related previous environmental permit case (matter ESAVI/34643/2019, decision 150/2020), the Southern Finland AVI granted a permit for spreading marl into the sea inside Finnish territorial waters with the purpose of sequestering phosphorus in the sediment. Marl is a side product of limestone mining, consisting of limestone, clay, and silt. The legal rule applied was section 27 of the environmental protection act on the general need for permit: a permit is required if there is a risk of pollution. The authorities did not consider section 18 of the environmental protection act that is dealing with dumping of material. The permit was granted for spreading 8 t of marl. The ministry advises that in future permits concerning the dissemination of materials into the sea, section 18 of the marine protection act is also acknowledged, and that the applicant explains a non-dumping purpose.

The operator must understand the specific characteristics of the case study area and describe them in the permit application. The Bothnian Bay is an oligotrophic sub-basin of the otherwise relatively eutrophic Baltic sea. Primary production in the Bothnian Bay is primarily limited by phosphorus, and there is a relatively high concentration of other nutrients available such as nitrate and silicate due to many rivers.^66^^,^^67^ The organic material is rich in refractory compounds such as humic acids that make the water yellow/brownish.^68^ Light does not penetrate very far into the water column, and it can be presumed that a modest increase in turbidity would not unacceptably alter the conditions for primary producers. However, one should consider the possible implications of particle additions for filter feeders and the functioning of gills.

Mercury, arsenic, and zinc concentrations are relatively high (typical ranges of 0.2–0.3 mg kg^−1^, 110–240 mg kg^−1^, and 70–250 mg kg^−1^ respectively) in the Bothnian Bay sediment, due to past industrial activities.^69^^,^^70^ Possible contaminations in the serpentinite gangue would be nickel, cobalt, copper, iron, and sulfate, and their exact loadings would need to be evaluated before starting OAE in the area. The ecotoxicity of nickel would be a major issue to be critically assessed.^71^ For environmental permitting, the applicant should present reliable estimates on the spreading dynamics, environmental fate, biological impact, and mitigation measures (Table 1).

**Table 1:** Estimating the impacts of nickel contaminations for permitting

Process	Description	Reference
Spreading dynamics	Nickel release is influenced by particle size, temperature, and pH.	Zhuang et al.^72^; Zhu et al.^73^
Environmental fate/impact	Nickel adsorbs onto sediments and forms complexes with organic and inorganic ligands.	Chen et al.^74^; Hutchins et al.^75^
Biological impacts	Nickel can act as a micronutrient at low concentrations but is toxic at high levels. The sensitivity to elevated nickel concentration is variable across species and trophic level.	Xin et al.^71^; Flipkens et al.^60^ Zheng et al.^76^
Mitigation strategies	Use of alkalization equipment/machinery (Figure 2) and monitoring programs can minimize impacts.	Zhuang et al.^72^; Zhu et al.^73^
Environmental safety standards	There is a need for safety standards considering local fauna.	Flipkens et al.^60^

As a conclusion, the LP or the Finnish law do not prohibit OAE as such. Finland abides by the precautionary approach as outlined in the LP, and a permit from the Northern Finland AVI would be needed. The Northern Ostrobothnia ELY would deem an environmental impact assessment (EIA) necessary, and most likely the EIA would require a trial period to provide measured results of effects. This trial period could take 4 to 5 years, during which the operators would study the impacts of nickel and other contaminants on marine species and habitats. The target of the Finnish authorities is to decide on environmental permit applications within 10 months from the date of submission, and under the law 1144/2022, priority is given in 2023–2026 to green projects, including CDR, carbon storage, and carbon use.

### Making OAE economically viable based on carbon market income

**Figure 5:**
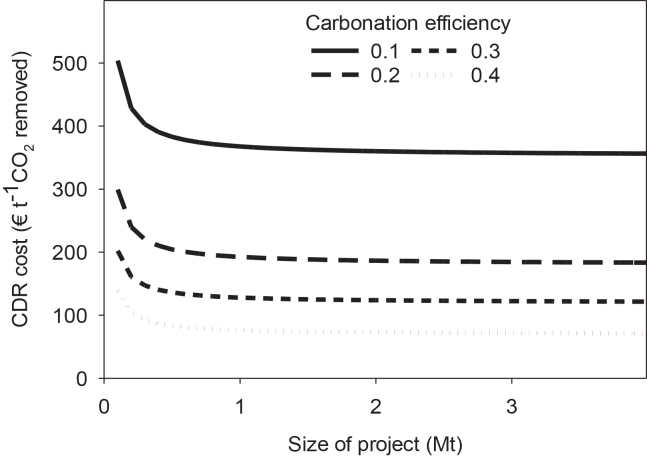
The CO_2_ removal (CDR) cost as a function of project size for ocean alkalinity enhancement (OAE) Assuming a milling size of 10 μm, and valuated for different carbonation efficiencies. Results are based on net CO_2_ removal.

**Figure 6:**
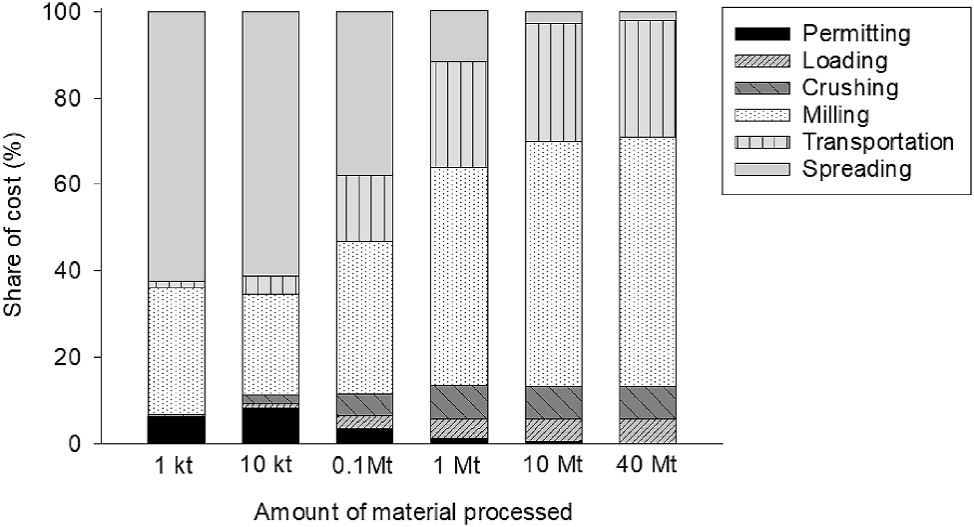
The share of different cost categories (by milling to 10 μm), depending on the project size The Hitura mine contains 5–6 Mt of suitable material and would have a cost structure positioned between the 1 and 10 Mt case. In order to achieve the lowest calculated cost of 90 € t^−1^ CO_2_ removed, a 40 Mt project is required.

Cost benefit calculation results show that the costs of OAE cannot completely be covered by the income through selling carbon credits. For a grain size of 10 μm, the estimated cost of CDR was 90–400€ t^−1^ CO_2_ removed, for projects exceeding 0.5 Mt (Figure 5). The project scale needs to be over 4 Mt to keep costs below 100 € t^−1^ CO_2_ removed, comparable to our Hitura case study with 5–6 Mt of suitable material, but it must be ∼40 Mt to get the minimum calculated price of 90 € t^−1^ CO_2_ removed (Supplementary Data file). The cost structure varies greatly between small and large projects. In the Mt scale, the milling costs make 50%–60% and transportation costs 30% of the total costs in the Hitura case (Figure 6). In smaller projects, the spreading costs become relatively more important in addition to cost related to loading, crushing, and permitting.

The carbonation efficiency is critical also for the economic viability of OAE. The lowest price of 90 € t^−1^ CO_2_ removed is achieved at a carbonation efficiency of 0.4, increasing to about 160 € t^−1^ CO_2_ removed at an efficiency of 0.33; at 0.1, costs clearly exceed current and projected CDR credit prices. The reported cost estimates represent central values; uncertainty associated with carbonation efficiency, milling energy intensity, and transport assumptions would further widen the cost interval. The scenario with a carbonation efficiency of 0.4 represents a best-case estimate, while efficiencies of 0.33 and 0.1 illustrate progressively lower-bound cases. In this scenario, the Hitura OAE project would be economically viable if the price of CDR credits exceeds 100 € t^−1^ CO_2_ removed. If milling to particle sizes smaller than 10 μm is required to achieve CO_2_ removal within a reasonable time frame, total costs would increase. To optimize milling and its cost, it would be important to better understand the carbonation efficiency as a function of the particle size range and temperature. As temperature affects carbonation efficiency, it will fluctuate throughout the seasonal cycle. Temperature was not explicitly considered in the present analysis; however, the calculations assumed spreading operations limited to eight months per year.

### Knowledge gaps of OAE and next steps

Taking an overarching look at OAE as a technology, there are several gaps of knowledge and technological barriers that must be overcome for OAE to play a role in climate change mitigation (Table 2). The basic chemistry is well understood, but there are uncertainties in the dissolution rates in different marine environments and the potential pathways of alkalinity loss e.g., by secondary precipitation of added minerals and biological alterations of alkalinity, which would reduce the CO_2_ removal potential. There is thus a need for more measurements of dissolution rates and the permanence of the carbon removal under realistic settings in various environment.

**Table 2:** The key knowledge gaps to address before large-scale OAE could be implemented

Knowledge Gap to be understood and Managed	Domain	Reference
**Engineering processes**		

Life cycle assessment of carbon footprint and identifying best suitable projects	engineering/environmental science	Foteinis et al.^19^
Reducing energy use and cost in fine grinding and milling	engineering/economic	Elbendari and Ibrahim^20^
Effective transport and dispersal	engineering	Renforth and Henderson^11^

**Oceanic processes: carbon cycle and marine ecology**		

Dissolution kinetics in different environments	chemistry	Geerts et al.^77^
Air-sea CO_2_ flux response to alkalinity enhancement	physical oceanography	He and Tyka^78^
CO_2_ permanence and carbon accounting	climate science, economic	Zhou et al.^79^
Regional biogeochemistry and ecosystem impacts	chemical/biological oceanography	Middelburg et al.^9^
Trace metal toxicity and ecological impacts	ecotoxicology	Guo et al.^80^

**Societal processes**		

Legal procedures, permitting	legal	Steenkamp and Webb^65^; Webb^27^; this paper
Economic viability, markets	economic	this paper
Monitoring, verification, reporting	technological, legal, economic	Halloran et al.^81^; Ho et al.^82^; this paper
Social license, stakeholder engagement	social science, legal	Satterfield et al.^83^

For calculating the carbon balance, there is a lack of input data for conducting a full LCA. The greening of energy production changes the calculation and could improve the outcome in terms of CO_2_ removal potential. To understand the effects on the pelagic ecosystems, small field trials and mesocosm experiments have been performed, but with much less work on higher trophic levels and benthic communities, which is still needed.

Environmental permitting and OAE carbon markets are in infancy. This creates uncertainties in the business model. Based on our analysis, our assessment is that OAE is currently at a technology readiness level (TRL) of 4–6.

Our LCA modeling quantified the value-chain greenhouse gas emissions of an OAE system based on serpentinite gangue from the Hitura mine and assessed its feasibility in terms of carbon balance, environmental impacts, legal permissibility, and economic viability. The results show that OAE can deliver substantial net CDR without extremely fine grinding. In the 10 μm milling scenario, the carbon emission factor was estimated to remain below 70 kg CO_2_e per tCO_2_ removed, indicating that life cycle emissions are unlikely to constitute a major barrier to the deployment of this technology.

From an environmental and biological perspective, potential impacts on marine ecosystems are central. While existing evidence suggests limited effects on primary producers under controlled conditions, uncertainties remain regarding species-specific responses especially at higher trophic levels and trace-metal release during mineral dissolution. In addition, alkalinity loss through secondary mineral precipitation or biological activity can reduce the net CO_2_ removal efficiency. These uncertainties highlight the need for site-specific ecological assessment and monitoring, particularly in coastal and semi-enclosed seas such as the Baltic sea.

From a legal and permitting perspective, OAE is not yet explicitly addressed under the London convention or the London protocol, leaving national authorities to interpret existing frameworks. In Finland, a comprehensive permitting process consistent with the precautionary principle can enable large-scale OAE if ecological risks and contaminant thresholds are carefully assessed. Nevertheless, legal uncertainty cannot be fully avoided, as permitting procedures are time-consuming and permits are typically temporary and revocable. Another regulatory challenge is the monitoring, reporting and verification of CO_2_ removal, as the carbon uptake due to OAE is not immediate but could stretch out over years.

The economic analysis indicates that the Hitura case study would not be economically viable at the Mt scale under prevailing carbon market conditions (mid-2020s). Even under the most favorable technical configuration assessed (10 μm particle size and a carbonation efficiency of 0.4), our best-case scenario with a bigger project size (40 Mt) than Hitura (5–6 Mt suitable material) gave a minimum price of 90 € t^−1^ CO_2_ removed (Supplementary Data file), exceeding European ETS allowance prices by 14% (∼70 € t^−1^ CO_2_ removed in 2025). In the Hitura case, using 5 Mt of gangue with carbonation efficiency 0.4 would remove 2 Mt of CO_2_. With an estimated cost of 100 € t^−1^ CO_2_ removed, it would incur a loss of 30 € t^−1^ CO_2_ removed, or 60 million € in total loss. To put this in perspective, it is 2.5-fold more than the 24 million € costs of the mine closure covered by the state of Finland. This suggests that OAE should be evaluated as a distinct CDR option that would likely need government support in some form (subsidies, tax credits etc).

Overall, the feasibility of OAE is strongly location- and technology-specific. Particle size, dissolution rates, energy systems, logistics, regulatory frameworks, and local ecological conditions jointly determine climate performance, environmental risks, costs, and permitting outcomes. Consequently, future OAE assessments should integrate LCA, site-specific ecological impact evaluation, legal analysis, and cost-benefit calculations to reduce uncertainty and support informed decision-making by operators and policymakers.

### Limitations of the study

This study is based on a hypothetical deployment scenario and several simplifying assumptions regarding carbonation efficiency, dissolution kinetics, transport logistics, environmental responses, and future economic conditions, and should be viewed as an order-of-magnitude assessment of this case study. The LCA relied partly on literature values and expert judgment rather than primary operational measurements, which in this case is not available. The carbonation efficiency of serpentinite in the cold, low-alkalinity Baltic sea is uncertain considering the seasonal cycle and environmental variability. The environmental assessment was limited to a desk-study analysis and did not include field experiments, hydrodynamic modeling, or direct ecological measurements, leaving uncertainties regarding long-term ecosystem impacts, such as trace-metal release possibly affecting benthic and higher trophic communities. The legal analysis reflects the current interpretation of Finnish and international regulations, which may evolve as governance frameworks for marine CDR develop. Similarly, the economic analysis is sensitive to uncertain future carbon prices, energy costs, monitoring requirements, and the development of robust monitoring, reporting, and verification systems for OAE. Finally, because the study focused on a single geological and oceanographic setting, the results are not directly transferable to other minerals or coastal ecosystems.

## Resource availability

### Lead contact

Requests for further information and resources should be directed to and will be fulfilled by the lead contact, Kristian Spilling (kristian.spilling@syke.fi).

### Materials availability

This study did not generate new unique reagents. All materials used are commercially available or previously published.

### Data and code availability

• The data is available as supplemental information.
• This paper does not report original code.
• Any additional information required to reanalyze the data reported in this paper is available from the lead contact upon request.

## Acknowledgments

This work was funded by internal funding at the 10.13039/501100013300Finnish Environment Institute (Syke seed money) and additionally supported by the Digital Waters Flagship project funded by the 10.13039/501100002341Research Council of Finland (former Academy of Finland), grant no 359250.

## Author contributions

Conceptualization, investigation, writing – original draft, writing – review and editing, A.L.-U.; conceptualization, methodology, investigation, writing – original draft, writing – review and editing, visualization, J.J.; conceptualization, methodology, investigation, writing – original draft, writing – review and editing, visualization, J.S.; writing – review and editing, S.L.; investigation, writing – original draft, T.M.; writing – review and editing, C.R.L.; writing – review and editing, U.R.; writing – review and editing, M.L.; conceptualization, investigation, writing – original draft, writing – review and editing, funding acquisition, project administration, K.S.

## Declaration of interests

Authors declare no competing interests.

## Declaration of generative AI and AI-assisted technologies in the writing process

During the preparation of this work the authors used ChatGPT and Google Gemini in order to improve the readability of some sections of the text, make literature searches and organize the reference list. After using this tool/service, the authors reviewed and edited the content as needed and take full responsibility for the content of the published article.

## STAR★Methods

### Key resources table

**Table:** 

REAGENT or RESOURCE	SOURCE	IDENTIFIER
**Deposited data**		

LCA and economic data	This paper, supplementary data file; Zenodo	https://doi.org/10.5281/zenodo.21244482

**Software and algorithms**		

SimaPro LCA software	https://simapro.com/	Version 10.3.0.1
Ecoinvent database	https://ecoinvent.org/	Version 3.11

**Other**		

Mineral composition of Hitura serpentine	Lavikko^53^	N/A

### Experimental model and study participant details

Omitted as our study does not involve biological models.

### Method details

#### Life cycle impact assessment

To quantify the system’s carbon footprint, material, energy and emissions inventories were characterized. Climate impacts were assessed using the global warming potential over a 100-year time horizon (GWP100), based on the IPCC AR6 (2021) characterization factors. The indicator is expressed in carbon dioxide equivalents (CO_2_e).

We followed the attributional life cycle assessment (ALCA) modelling framework to create the Life cycle inventory (LCI).^84^ This suited our objectives as our study is retrospective and focuses on hypothetical “what-if” scenarios. We do not anticipate any significant consequential impacts that would need to be considered in a consequential LCA analysis; the use of mine gangue for OAE is not expected to result in any major changes or unintended consequences in the wider system. Since the case is hypothetical, we did not have access to primary data for the life cycle inventory. Therefore, our analysis is based on a combination of literature review and expert estimates. Key components considering OAE is the impact factor which is used to translate materials and activities into CO_2_ emissions equivalents CO_2_e, and the carbonation efficiency, which is the amount of CO_2_ removed per material used (t CO_2_ removed / t OAE material used).

The study represents a streamlined form of LCA, aiming at a quick quantification of climate impacts (carbon footprint) of the studied system. It is important to note that both the data used for the background system and the assumptions made for the foreground system (e.g., the amount of electricity consumed by a mill) are inherently uncertain. To address this issue and to ensure that our conclusions are not misleading, we conducted a scenario analysis and utilized a Monte Carlo simulation to investigate the sensitivity and uncertainty of the analysed system. By taking this approach, we gain a better understanding of the range of possible outcomes and make more informed decisions based on our findings. For the background data, the ecoinvent 3.11 database was used. The use of the recent version of the background database poses a limitation regarding some datasets which represent industrial processes that have evolved since 2015. This includes, for example, the Finnish electricity consumption mix, which has decarbonised in recent years significantly.

The model was constructed in the SimaPro LCA software (version 10.3.0.1). The model was set up for scenarios by default as follows. The on-site transport is done by a conveyor belt. Ecoinvent background data was used for the excavator and the conveyor belt operations. Transport from the mine to the harbor as well as the sea transport were modelled based on the cost benefit analysis described below.

#### Cost-benefit analysis

We conducted a cost-benefit analysis^85^ for implementing OAE at the selected case study location. All cost estimates exclude value-added tax (VAT), and an exchange rate of 0.89 USD/EUR was used for currency conversion. Monetary values from original sources were adjusted to 2022 euros using the Finnish cost-of-living index.^86^

### Quantification and statistical analysis

#### Life cycle impact assessment

The LCA model was constructed according to the following LCI data.

##### Loading of serpentine gangue at hitura mine

The loading serpentine gangue at the mine was modeled as two alternative options:
  A . Literature-based. Hajji^87^ states the productivity of an excavator with the bucket size 3 cy to be 134.61 m^3^ h^-1^. This value was used in the baseline scenario and as the upper estimate in the uncertainty analysis, with 100 m^3^ h^-1^ on the lower limit. To load 1 kg of serpentine gangue, we calculated 4.27E-6 h of the operation of the respective excavated needed.
  B . Ecoinvent process *Excavation, hydraulic digger {GLO}| market for excavation, hydraulic digger | Cut-off, U.* The functional unit of the ecoinvent unit process is m^3^. The density of a rock pile was assumed to be 1.74E3 kg m^-3^. Thus, 0.000575 m^3^ represents 1 kg of serpentine gangue.

##### Transport of serpentine gangue within the hitura mine

The transport of serpentine gangue within the mine is modelled as two alternative options.
  A. Conveyor belt. The ecoinvent unit process *Transport, freight, conveyor belt {GLO}| transport, freight, conveyor belt | Cut-off, U* has been modified by switching the electricity mix from GLO to FIN. The distance of the conveyor belt transport was assumed to be 600 m.
  B. Dump truck. The operation of a dump truck was estimated as follows. Fuel consumption is between 50 and 100 l of diesel per 1 hour of operation (baseline scenario 75 l; i.e., 63.8 kg). Corresponding direct CO_2_ emissions are 201 kg CO_2_. Lifespan of a dump truck is 10 years (assuming operation 24/7). To model dump truck transport, it was assumed that the productivity of a dump truck is 58.924 m^3^ h^-1^ and that the density of a rock pile is 1.74E3 kg m^-3^.

##### Crushing at hitura mine

The crushing process represents crushing of serpentine gangue to gravel of the size 1 cm (10000 μm). The default electricity consumption of the crusher is based on Landfield and Karra^88^; 0.717 kWh t^-1^). The range for the uncertainty analysis is based on Tosun and Konak^89^; 0.4-1.5 kWh t^-1^).

The manufacturing of a crusher was estimated according to the inventory based on Landfield and Karra^88^ where a crusher has a total lifetime production of 56 750 000 t (life span: 25 years, crusher throughput capacity measured at the plant: 454 t h^-1^, crusher production: 5000 h a^-1^.

##### Milling at hitura mine

The electricity consumption of the mill was modelled according to the Bond Work Index (*Wi*). The Index represents the required energy in kWh required to reduce a one metric tonne of given material from theoretically infinite feed size to 80% passing 100 μm. For the baseline scenario we used the value of 20 kWh t^-1^, and a range between 10 and 30 kWh t^-1^ for the uncertainty analysis. The electricity consumption was calculated according to the following function:

(Equation 1) W=10Wi((1/√P)−(1/√P))

where Wi stands for the Bond Work Index, F for feed and P for product. The parameters F and P are expressed in μm and defined as 10000 μm for the feed and 10 for the default product (subject of scenarios and sensitivity analysis). The crusher inventory was used as a proxy for the mill manufacturing (see above).

##### Transport of the material to the harbour

The transport of the material from the Hitura mine to the Kokkola harbour is modelled as truck transport over the distance 100 km in two optional ways.
  A. CBA calculation. Both trips to the harbor and the empty return trips are considered (200 km in total). The fuel consumption (*Diesel, low-sulfur {RER}| market group for diesel, low-sulfur | Cut-off, U*) of the truck based on the CBA calculation is 0.00425 kg of diesel tkm^-1^. The corresponding direct CO_2_ emissions are approximately 13.56 g CO_2_ tkm^-1^.
  B. Ecoinvent process *Transport, freight, lorry* >*32 metric tonne, EURO6 {RER}| market for transport, freight, lorry* >*32 metric tonne, EURO6 | Cut-off, U.* The ecoinvent unit process is modelled to include empty return trips. Thus, only the distance of 100 km is considered.

##### Transport of the material from the harbour and its dispersion in the EEZ

The transport from Kokkola harbour to the Finnish EEZ is modelled in two optional ways.
  A. Fuel consumption of the pontoon per metric tonne of material based on the CBA calculation: 1.39 kg of *Petrol, low-sulfur {Europe without Switzerland}| market for petrol, low-sulfur | Cut-off, U.* The number was derived by considering the daily fuel consumption of a pontoon (124 l), pontoon’s capacity (64.8 t d^-1^) and petrol density (0.725 kg l^-1^). The corresponding direct CO_2_ emissions are approximately 4.37 kg CO_2_.
  B. Modified ecoinvent process of *Transport, freight, inland waterways, barge tanker {RER}| processing | Cut-off, U.* The barge transport considered in our model is sea transport, not inland waterways transport, thus the construction of a canal has been removed. The distance from the harbour to international waters is 22 km (12 nautical miles).

To understand the overall uncertainty of the results, we performed a Monte Carlo simulation for each of the three scenarios. Monte Carlo analysis is a statistical method used to estimate the uncertainty of a system by simulating it many times with random inputs. We applied 5000 iterations. The result of the Monte Carlo simulation is a probability distribution of possible outcomes and the likelihood of each outcome occurring. Each data input in the background ecoinvent database is defined as an uncertain parameter with lognormal distribution. In the foreground system, i.e., the model created by us, we defined the uncertainty for most variables (e.g., electricity consumption of the crusher, the Bond Work Index for milling operations) as a uniform distribution, which essentially is a range of equally likely values. Uncertainties of several parameters is defined as triangular distribution. When running the Monte Carlo simulation, all parameters, including those in the background system are varied. Thus, the results of the uncertainty analysis illustrate global uncertainty of the model and the data it is built on. The uncertainties are listed in the supplemental information (Table S1).

To study the sensitivity of modelling choices on results, we performed a sensitivity analysis for the scenarios producing the 10 μm product. We modified the variables that define what type of unit processes or background data is used in the model. The sensitivity analysis was done for the following parameters: the excavator unit process inventory, the type of transport within the mine, the type of inventory the truck transport from the mine to the harbor, and the type of inventory for the sea transport (See Table S2 in the supplemental information). Results of the sensitivity analysis are presented side-by-side in absolute values.

**Figure 7:**
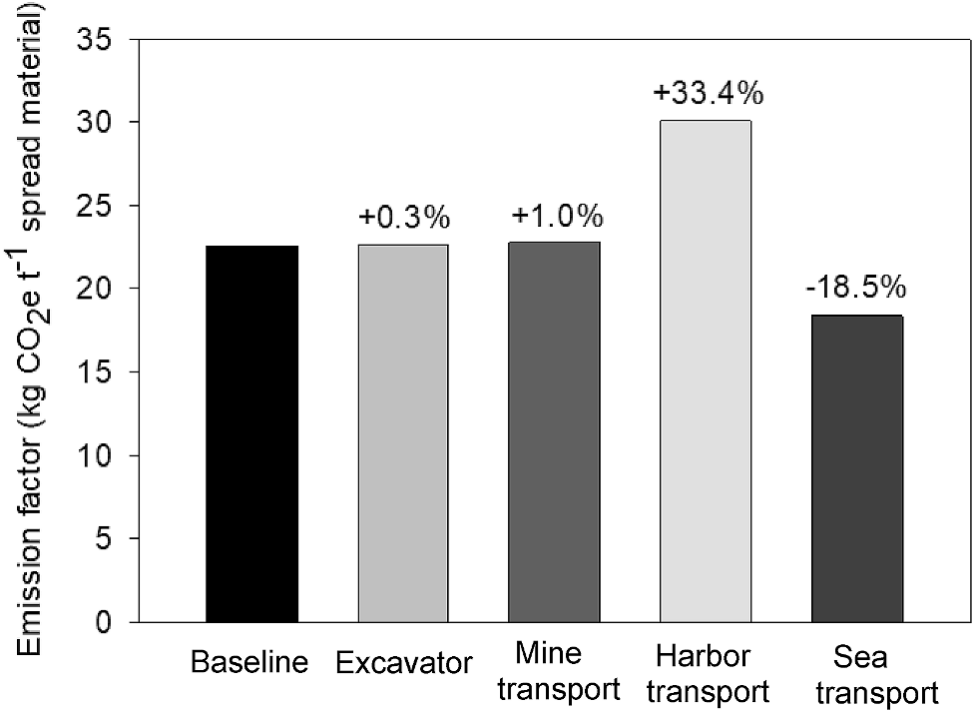
Sensitivity analysis (SA) of the emission factor of different scenarios with spreading of 10 μm particles

The results of the sensitivity analysis performed for the 10 μm scenario show variation of results between -18.5% and +33.4% (Figure 7). Because the contribution of excavator operations is very small already in the default setup of the 10 μm scenario (baseline), the choice of the background data does not affect results much (+0.3%). The assumption regarding mine logistics, i.e., whether a conveyor belt or a dump truck is considered, makes only a small difference, too. The use of a dump truck increases the impacts of the analysed system by 1% compared to a conveyor belt. The modelling choice of truck transport from the Hitura mine to the Kokkola harbor has the biggest impact on results. Truck transport contributes to 18% of scenario’s impacts. The impacts would increase by 33.4%, if transport was modelled in the standard way based on the ecoinvent background data and their impacts per tonne-km. The load factor of the ecoinvent transport process, however, is low as it represents a European average load factor for different kinds of cargo. Lastly, the carbon footprint would decrease by 18.5% if instead of using the pontoon inventory done for the purposes of the CBA we consider a standard river barge dataset obtained from the ecoinvent database. This is because a pontoon and a barge are very different types of vessels, the first one being small and inefficient while the latter one being much larger and more efficient. The pontoon transport contributes to 26% of total impacts of the 10 μm scenario while the barge transport would contribute to only 7%, thus the choice of the background data is important. The choice of transport background data would be even more important for the 100 μm and 1000 μm scenarios where transport processes dominate the impacts. For example, the contribution of truck transport to the overall impacts in the 1000 μm scenario is 36%. If the ecoinvent transport process was used, the impacts would increase by 2/5.

#### Cost-benefit analysis

The full economic costs of the physical OAE process were categorized into the following cost categories: permitting and verification (P), loading (L), crushing (C), milling (M), transporting to harbor from the mine (T) and spreading (S) (Figure 2). All these costs (in €) are functions of the metric tonnes of material handled (t_m_).

The benefit, B(t_CO2_) is the monetary value (€ / t CO_2_ removed), which in this case study is paid for by a buyer of the OAE service as a carbon offset. For the project to be economically viable, the benefit must exceed the total costs:

(Equation 2) $$B\left(t_{CO2}\right)>\left[P\left(t_{m}\right)+L\left(t_{m}\right)+C\left(t_{m}\right)+T\left(t_{m}\right)+S\left(t_{m}\right)\right]/I$$

Where P(t_m_) is the permit cost, L(t_m_) is the loading cost, M(t_m_) is the milling cost, T(t_m_) is the cost for transportation, S(t_m_) is the spreading cost, and I is the impact factor = t_m_ / t_CO2_ (the inverse of the carbonation efficiency). The gross carbon dioxide removal is given by CDR_gross_= t_m_ / I, and the net removal by CDR_net_ = CDR_gross_ – (t_m_ ∗ EF), where EF is the emission factor (t CO_2_e t_m_^-1^ mineral spread; Figure 3). A realistic carbonation efficiency in our case is up to ∼0.4 for 10 μm particle size (I = 2.5) and <0.1 for 1000 μm particle size.^26^^,^^90^

The permit costs, P(t_m_), consist of environmental permitting and compliance costs. This includes the application fee for permits, monitoring (including GHG and environmental monitoring), reporting, and carbon removal verification and reporting. In Finland, the cost for a full EIA for operators has ranged from 0.001 % to 1 % of the total project cost, from tens of thousands to hundreds of thousands of euro.^91^ We assess the EIA before the start of large-scale operations for our OAE project to cost 50 000 - 200 000 €. The permit application including permit fee is estimated to cost 50 000 - 150 000 € per project. Due to the novelty of OAE activity, we expect the permit authority to require post-operations monitoring for five years. Biota monitoring would need to be conducted including community composition and relevant biomarkers. Natura2000 evaluation under the EU Habitats Directive would not be necessary at the location. Monitoring and reporting costs are estimated as 25 000 - 100 000 € per project. Compliance costs are associated with the verification and validation of carbon removal using required standards, typically ISO 14064. Similarly to how the operator must describe the environmental impacts to the permitting authority, it must describe the carbon capture and sequestration that it wants to be verified. In voluntary carbon offset markets, the OAE service could be offered to be sold for example via the GoldStandard site. OAE could find markets alone or as part of a bundle of carbon removal technologies. The estimated cost to verify a project at the carbon offset market would be minimum 8000 €.^92^ As the OAE technology is new, we evaluate this cost to be higher at 30 000 €. Our estimate assumes a standardized way of calculating CO_2_ removal based on the quantity and quality of the mineral used and the environmental conditions. Verification of the CO_2_ removal on a case-by-case basis at the site would require considerably more monitoring effort. In conclusion, it is hard to assign P cost directly as a function of materials processed as there are some fixed costs involved, although the size of the project clearly matters. We estimate P to be 100 000 € for a small 1 kt project, 200 000 € for a medium-size 50 kt project, and 500 000 € for a large 5 Mt project. These assumptions lead to the following approximation regarding the permitting costs:

(Equation 3) $$P\left(t_{m}\right)=47496\;\ln\left(t_{m}\right)\;–\;258203$$

where ln(t_m_) is the natural logarithm of tonne mineral spread.

The loading, L(t_m_), cost is based on Ozcelik^93^ and involves moving the material within the mine to a truck. The cost function can be presented as:

(Equation 4) $$L\left(t_{m}\right)=2\;t_{m}$$

Crushing, C(t_m_), includes crushing rock of 100 mm (F_80_) to 6 mm (P_80_), based on Eloranta^94^ prices, the cost function can be presented as:

(Equation 5) $$C\left(t_{m}\right)=3\;t_{m}$$

Milling costs include the capital and operation costs based on the model developed by Sayadi et al.^95^ The milling cost function, M(t_m_), can be presented as:

(Equation 6) $$M\left(t_{m}\right)=[1.13\;*[(19700*W^{\wedge }0.664/t_{m}+1.12*W^{\wedge }0.73]]*t_{m}$$

where W, the predicted mill energy consumption, based on Bond's index,^96^ the common form is given in Equation 1, where the work index Wi (kW h t_m_^-1^) used here was Wi=17.1 (granite). Rock with size 6000 μm (F) was milled to the required size (P) of 10 μm.

Transportation cost, T(t_m_) is based on the distance from the mine to the harbor, which is 100 km, and the unloading of trucks is included. Based on the Finnish market price, the cost estimate function is:

(Equation 7) $$T\left(t_{m}\right)=10\;t_{m}$$

The cost of offshore spreading is estimated assuming the use of a dry bulk carrier. The estimate includes fuel costs, port dues, capital costs, costs related to mixing powder with water, and other operational expenses associated with spreading operations.^97^ According to Caserini et al.,^98^ spreading rates of up to 1 t_m_ s^-1^ can be achieved using a dry bulk carrier. The core term 1349.6 A(t_m_)^0.2933^ is an empirically fitted cost function derived from daily cost of selected bulk carrier and the amount of spread material.^97^ We assumed that the project operates for 260 working days per year (no winter operations), of which 130 days are allocated to loading the carrier and 130 days to spreading the material. Any required modification of the carrier for spreading operations, as well as extended loading durations, are accounted for using a spreading adjustment factor D. The spreading cost function is defined as:

(Equation 8) $$S\left(t_{m}\right)=1349.6*A\left(t_{m}\right)ˆ0.2933\;*\;Wd*D$$

where A(t_m_) = t_m_ / 130 here, and represents the amount of material in metric tonnes spread per active spreading day. W_d_ is the number of annual spreading days (W_d_ = 130 here). D is a dimensionless factor that increases costs to reflect vessel modification requirements or longer loading times (D = 2 here).

The minimum achievable cost is constrained by the operational scale of dry bulk carriers, which have typical capacities of 200 – 300 kt per voyage. For keeping costs low, it is important to utilize trucks and vessels at maximum capacity, if e.g. vessel capacity is underutilized the spreading costs per metric tonne increase. Under the assumptions in Equations 2–9, we find that project scales needs to be over 4 Mt to keep costs below 100 € t^-1^ CO_2_ removed, comparable to our Hitura case study with 5-6 Mt of suitable material, but it should be ∼40 Mt to get the minimum calculated price of 90 € t^-1^ CO_2_ removed.

## Supplemental information

Document S1. Tables S1 and S2

Data S1. The LCA data and economic analysis
