# Supplementary material for: Tackling the technological, environmental, legal, and economic challenges of ocean alkalinity enhancement: A case study from the Baltic sea
Source: iScience. 2026 Jul 30;29(8):116928. doi: 10.1016/j.isci.2026.116928 (PMC13452245; doi:10.1016/j.isci.2026.116928)
Supplement: Supplementary file 1 [file mmc1.pdf]

iScience, Volume *29*

Supplemental information

Tackling the technological, environmental, legal, and economic challenges of ocean alkalinity enhancement: A case study from the Baltic sea

Anu Lähteenmäki-Uutela, Jáchym Judl, Jari Silander, Sonja Lavikko, Tuukka Mäkelä, Carolin Regina Löscher, Ulf Riebesell, Markus Latvala, and Kristian Spilling

Supplementary material

Uncertainties of model parameters

Table S1. Uncertainty values for model parameters

Process	Parameter	Default value	Unit	Type of uncertainty	Min	Max	Note
**Project parameters**	**Density, gasoline**	0.725	kg/l	Uniform	0.7	0.75	
	**Density, serpentine**	2900	kg/m^3	Uniform	2500	3300	
	**Serpentine gangue diameter**	12.5	cm	Uniform	10	15	The diameter of serpentine gangue at the Hitura mine.
**serpentinite gangue, rocks, loading**	**Excavator productivity**	134.61	m^3/h	Uniform	100	134.61	
	**Type of excavator**	1	-	Uniform	0	1	Switching parameter. If <= 0.5, then ecoinvent's excavator unit process is used, if = > 0.5, then literature-based excavator inventory is used.
**serpentinite gangue, rocks, at crusher**	**Type of transport**	0	-	Uniform	0	1	Switching parameter. If <= 0.5, then conveyor belt is used, if = > 0.5, then dump truck is used.
**serpentinite, milled**	**Work index (Wi)**	20	kWh/t	Triangle	10	30	The energy required in kWh/t to reduce a given material from theoretically infinite feed size to 80% passing 100 microns (0,001 cm)<br><br>Bond Work Index table https://www.911metallurgist.com/blog/table-of-bond-work-index-by-minerals
**milled product, at harbor**	**Type of truck transport**	0	-	Uniform	0	1	Switching parameter. If <= 0.5, then the truck based on the CBA analysis is used, if = > 0.5, then the ecoinvent truck dataset is used.
**milled product, at sea**	**Type of sea transport**	0	-	Uniform	0	1	Switching parameter. If <= 0.5, then the pontoon based on the CBA analysis is used, if = > 0.5, then the ecoinvent barge dataset is used.
**dump truck, operation**	**Fuel consumption, dump truck**	75	l/h	Uniform	50	100	Fuel consumption set to be between 50 and 100 liters of diesel per 1 hour of operation.
**dump truck, materials**	**Scaling parameter**	0.9	-	Uniform	0.8	1	Scaling parameter used to vary the amounts of materials used to manufacture a dump truck. The default amounts of materials are multiplied by this parameter. Per 1 truck, we consider the need of 1 t of rubber, 12 tons of steel, 200 kg of plastics, and 50 kg of glass.
**operation, excavator (400 hp, 3 cy bucket size)**	**Operation service life**	10000	h	Lognormal	SD 1.62		Pedigree matrix (4,5,5,2,1,na), According to the ecoinvent, the average service life of a hydraulic digger is 10000 hours. The parameter is used to allocate the digger's manufacturing to one hour of operation,
**pontoon transport (per hour of operation)**	**Fuel consumption, pontoon**	16	l/h	Uniform	12	19	Fuel consumption of the pontoon based on the CBA calculation: motor 10-17 l/h, diesel generator 2 l/h
	**Material spreading speed**	10800	kg/h	Uniform	2520	34560	How much material is spread from the pontoon per hour.
**pontoon transport (per tonne of OAE)**	**Fuel consumption, pontoon**	124	l/day	Uniform	96	152	Values based on the CBA calculation: motor 10-17 l/h, diesel generator 2 l/h, 8 hours
	**Material spreading speed**	64.8	t/day	Uniform	10.08	241.92	How much material is spread from the pontoon per day, min = 10,080 t/h, max = 241,92 t/h (default 64,8 t/day)
**rock crusher, operation**	**Energy, crusher**	0.717	kWh/t	Triangle	0.4	1.5	A rough range based on the paper by Tosun and Konak (2014)
**truck transport, 50 t payload**	**Fuel consumption, truck**	25	l/100 km	Uniform	20	30	Based on the CBA spreadsheet, Scania R 500 V. 2008

Table S2. Sensitivity analysis: baselines and scenario modifications

<table>
<tr><th>Sensitivity analysis</th><th>Baseline</th><th>Scenario modification</th></tr>
<tr><td>excavator</td><td>Excavator operation based on the ecoinvent dataset: Excavation, hydraulic digger {GLO}| market for excavation, hydraulic digger | Cut-off, U</td><td>Literature-based inventory for an excavator based on Hajji (2014)</td></tr>
<tr><td>mine transport</td><td>Rock transport within the mine on conveyor belt (ecoinvent data): Transport, freight, conveyor belt {FI}| transport, freight, conveyor belt | Cut-off, U (modified version of the GLO dataset, using Finnish electricity grid mix)</td><td>Dump truck used instead of the conveyor belt. Inventory based on Hajji (2014)</td></tr>
<tr><td>harbor transport</td><td>Transport to the harbor modeled based on the cost benefit analysis of this study</td><td>Truck transport process from the ecoinvent database: Transport, freight, lorry, >32 metric ton, diesel, EURO 6 {RER}| market for transport, freight, lorry, >32 metric ton, diesel, EURO 6 | Cut-off, U</td></tr>
<tr><td>sea transport</td><td>Sea transport modelled based on the cost benefit analysis of this study for a small pontoon.</td><td>Barge sea transport, ecoinvent dataset: Transport, freight, inland waterways, barge tanker {RER}| processing | Cut-</td></tr>
</table>

		off, U (modified version where the construction of the canal is removed)

9
